# Secretory IgA reduced the ergosterol contents of *Candida albicans* to repress its hyphal growth and virulence

**DOI:** 10.1007/s00253-024-13063-z

**Published:** 2024-02-29

**Authors:** Jiannan Wang, Jiawei Shen, Ding Chen, Binyou Liao, Xi Chen, Yawen Zong, Yu Wei, Yangyang Shi, Yaqi Liu, Lichen Gou, Xuedong Zhou, Lei Cheng, Biao Ren

**Affiliations:** 1 State Key Laboratory of Oral Diseases & National Center for Stomatology & National Clinical Research Center for Oral Diseases, West China School of Stomatology, Sichuan University, Chengdu, 610041 Sichuan China; 2 Department of Operative Dentistry and Endodontics, West China School of Stomatology, Sichuan University, Chengdu, 610041 Sichuan China; 3 Department of Pediatric Dentistry, West China Hospital of Stomatology, Sichuan University, Chengdu, 610041 Sichuan China

**Keywords:** Fungal infection, Secretory immunoglobulin A, Hyphal development, Ergosterol biosynthesis, Virulence

## Abstract

**Abstract:**

*Candida albicans*, one of the most prevalent conditional pathogenic fungi, can cause local superficial infections and lethal systemic infections, especially in the immunocompromised population. Secretory immunoglobulin A (sIgA) is an important immune protein regulating the pathogenicity of *C. albicans*. However, the actions and mechanisms that sIgA exerts directly against *C. albicans* are still unclear. Here, we investigated that sIgA directs against *C. albicans* hyphal growth and virulence to oral epithelial cells. Our results indicated that sIgA significantly inhibited *C. albicans* hyphal growth, adhesion, and damage to oral epithelial cells compared with IgG. According to the transcriptome and RT-PCR analysis, sIgA significantly affected the ergosterol biosynthesis pathway. Furthermore, sIgA significantly reduced the ergosterol levels, while the addition of exogenous ergosterol restored *C. albicans* hyphal growth and adhesion to oral epithelial cells, indicating that sIgA suppressed the growth of hyphae and the pathogenicity of *C. albicans* by reducing its ergosterol levels. By employing the key genes mutants (*erg11Δ/Δ*, *erg3Δ/Δ*, and *erg3Δ/Δ erg11Δ/Δ*) from the ergosterol pathway, sIgA lost the hyphal inhibition on these mutants, while sIgA also reduced the inhibitory effects of *erg11Δ/Δ* and *erg3Δ/Δ* and lost the inhibition of *erg3Δ/Δ erg11Δ/Δ* on the adhesion to oral epithelial cells, further proving the hyphal repression of sIgA through the ergosterol pathway. We demonstrated for the first time that sIgA inhibited *C. albicans* hyphal development and virulence by affecting ergosterol biosynthesis and suggest that ergosterol is a crucial regulator of *C. albicans*-host cell interactions.

**Key points:**

*• sIgA repressed C. albicans hyphal growth*

*• sIgA inhibited C. albicans virulence to host cells*

*• sIgA affected C. albicans hyphae and virulence by reducing its ergosterol levels*

**Supplementary Information:**

The online version contains supplementary material available at 10.1007/s00253-024-13063-z.

## Introduction

*Candida albicans* is the most common conditional pathogenic fungus from the human microbiome. It can colonize in different niches of human body, such as mouth, skin, and gastrointestinal and vaginal tracts (Belvoncikova et al. 2022; Lopes and Lionakis 2022; Proctor et al. 2023). *C. albicans* can also cause superficial fungal infections, such as oral candidiasis, and systematic fungal infections, such as candidemia, especially in the immunocompromised populations, including organ transplantation patients, cancer patients during radiation or chemotherapy, HIV-infected patients, and patients with gastrointestinal surgery (Kullberg and Arendrup 2015; Pappas et al. 2018; Talapko et al. 2021; Thomas-Rüddel et al. 2022). During its infectious process, *C. albicans* hyphal development is considered as the most important virulence factor, contributing to the damage to host cells and tissues and interactions with host immune cells, proteins, and molecules (Chen et al. 2020a; Day and Kumamoto 2023; Kumamoto et al. 2020; Zhou et al. 2021a).

Secretory immunoglobulin A (sIgA) is a significant antibody isotype from the host mucosal immune system and plays a critical role in mediating the pathogenesis of several infections (Belkaid and Harrison 2017; Hooper and Macpherson 2010; Weis and Round 2021). sIgA is crucial in controlling *C. albican*s communism and pathogenicity. sIgA is capable to decrease the *C. albicans* attachment to dental material in vitro (Elguezabal et al. 2004; Holmes et al. 2002; San Millán et al. 2000; Umazume et al. 1995). Intestinal IgA could bind to *C. albicans* cells to downregulate the adhesins, such as *ALS1* and *ALS3*, to promote its fitness (Ost et al. 2021). Reduced gut sIgA reactivity to *C. albicans* hyphae increased the hyphal forms in Crohn’s disease patients (Doron et al. 2021a) and the absence of sIgA caused an overgrowth of *C. albicans* and microecological imbalance in the intestinal tract, while sIgA decreased the proportion of *C. albicans* that adhered and invaded to intestinal cells (Moreno-Sabater et al. 2023). In the oral cavity, salivary secretory IgA can recognize and bind to *C. albicans* phosphoglycerate kinase (an ATP-producing and energy-generating enzyme) and fructose bisphosphate aldolase (participating in cell wall biosynthesis) (Calcedo et al. 2012). sIgA is also capable to inhibit the adhesion and penetration of *C. albicans* into oral epithelial cells in oropharyngeal candidiasis mouse (Millet et al. 2020). Although the essential roles of sIgA for regulating *C. albicans* colonization and pathogenesis in different host niches have been proven, the effects and mechanisms that sIgA directly acts on *C. albicans* to affect its morphological transformation and virulence are still unclear.

Ergosterol, the key component of the *C. albicans* cell membrane, is essential to maintain cell membrane heterogeneity, prevent water from penetrating, and keep the plasma membrane’s integrity, rigidity, and fluidity (Abe et al. 2009), while itself and its biosynthesis are the targets of current clinical antifungal drugs, including polyenes and azoles, respectively (Lee et al. 2021). It is also highly related to *C. albicans* hyphal development (Lv et al. 2016). The inhibitions of some *C. albicans* genes of the ergosterol biosynthesis pathway, such as *ERG1*, *ERG11*, *ERG12*, and *ERG13*, significantly repressed the hyphal development (O'Meara et al. 2015), and azoles could also decrease *C. albicans* hyphal formation by targeting at the ergosterol biosynthesis (Kartsonis et al. 2003). However, whether the ergosterol and its biosynthesis are associated with the actions of sIgA on *C. albicans* is still unclear.

In this study, we directly treated *C. albicans* with different doses of sIgA and investigated its effects and mechanism on the hyphal development and virulence, including adhesion and cell damage to oral epithelial cells. Our results revealed that sIgA affected the ergosterol biosynthesis pathway and reduced the ergosterol contents to repress *C. albicans* hyphal growth, adhesion, and damage to host cells. We identified the target pathways of sIgA on *C. albicans* to regulate its pathogenesis and highlight that *C. albicans* ergosterol is important for its virulence and interactions with host immune proteins.

## Materials and methods

### Chemicals

The polyene agent filipin (Cat. S27675, Shanghai Yuanye Bio-Technology Co., Ltd., Shanghai, China) was dissolved in dimethyl sulfoxide (DMSO, Cat. 196055, MPbio, Shanghai, China). Ergosterol (Cat. E808836, 97% purity, MACKLIN, Shanghai, China) was dissolved in isopropanol. sIgA and IgG were purchased from BIO-TC (Cat. Ns04-12 (sIgA); Ns04-08 (IgG), Luoyang Baitaike Biotechnology Co., Ltd, Luoyang, China). The compounds were stored at − 20 °C.

### Strains and media

Supplemental Table S1 listed all the strains and mutants employed in this study. All the *C. albicans* strains were maintained on YPD plates at 35 °C overnight as describe previously (Chen et al. 2020b; Zhou et al. 2018).

### *C. albicans* hyphal formation measurement

The hyphal development was analyzed as described previously (Lemberg et al. 2022; Liang et al. 2023). Single *C. albicans* colony from YPD plates was picked into RPMI 1640 (Gibco, Shanghai, China) liquid medium with the final concentration at 5 × 10^4^ colony-forming unit (CFU) /ml. The fungal cells were treated by 50 and 100 μg/ml of sIgA or immunoglobulin G (IgG) as control, and the cultures were incubated at 37 °C, 200 rpm for 2 or 4 h. After that, the hyphal cells in the culture were observed by a phase-contrast microscope (DMi8, Leica, Shanghai, China). The distribution of hyphal length was determined from more than 200 fungal cells in each group by utilizing ImageJ software (Version 1.8.0.172, National Institutes of Health, America).

### Epithelial cell adhesion assay

*C. albicans* adhesion to oral epithelial cells was performed as described in the previous study (Zhou et al. 2018). Briefly, human oral keratinocytes (HOK) were cultured in 24-well plates with 2 × 10^5^ cells/ml in each well and incubated at 37 °C, 5% CO_2_ overnight. *C. albicans* (2 × 10^5^ cells/ml) was preincubated with 100 μg/ml sIgA or IgG for 30 min. The *C. albicans* treated with phosphate buffered saline (PBS) was served as control. Then, *C. albicans* was collected and rinsed with PBS for three times and added into the HOK cell culture and incubated for another 60 min. The planktonic *C. albicans* cells were washed and HOK cells were digested with trypsin. Then, the cells were collected and homogenized and spread on YPD plates. The YPD plates were then incubated at 35 °C overnight, and the CFU was calculated.

### Cell damage assay

The Roche cytotoxicity detection kit^plus^ (Roche, Shanghai, China) was used to perform the cell damage assay (Zhou et al. 2018, 2021b). Briefly, the HOK cells (2 × 10^5^ cell/ml) were cultured in 96-well plates using Dulbecco’s Modified Eagle’s Medium (DMEM, Gibco, Shanghai, China) with 10% fetal bovine serum (FBS, Gibco, Shanghai, China) for 24 h. The supernatant was replaced by fresh DMEM medium with *C. albicans* (2 × 10^7^ CFU/ml), and then, the cultures were treated with or without sIgA or IgG (100 μg/ml) at 37 °C, 5% CO_2_ for 8 h. The lactate dehydrogenase (LDH) activity was assessed according to the manufacturer guidelines.

### Filipin binding assay

The binding affinity between filipin and *C. albicans* cells was measured as described previously (Ren et al. 2014; Zhu et al. 2021). *C. albicans* were cultured in RPMI 1640 medium at a final concentration of 5 × 10^4^ CFU/ml and treated by sIgA (0, 50, and 100 µg/ml) for 2 and 4 h. *C. albicans* cells were collected and rinsed by PBS and then treated with 20 µg/ml filipin at 37 °C, 200 rpm for 30 min. The cultures were then collected and washed by PBS. SpectraMax iD5 reader (Molecular Devices, LLC., San Jose, America) with excitation at 340 nm and emission at 480 nm was employed to measure the fluorescence.

### RNA sequencing and analysis

The RNA sequencing was implemented to invest the mechanisms of sIgA (Wei et al. 2023; Yawen et al. 2022). *C. albicans* (5 × 10^6^ CFU/ml) was treated with sIgA in RPMI 1640 medium (0 µg/ml, 50 µg/ml, and 100 µg/ml) at 37 °C, 200 rpm for 2 and 4 h. *C. albicans* cells were collected and stored in liquid nitrogen. The RNA sequencing was conducted by Shanghai OE Biotech Co., Ltd., Shanghai, China. The sequencing data were accessible online (https://www.ncbi.nlm.nih.gov/sra/PRJNA 1016252).

### Real-time PCR assay

The expressions of ergosterol biosynthesis genes were measured by RT-PCR assay. *C. albicans* was treated by sIgA (0, 50, and 100 µg/ml) for 2 and 4 h. *C. albicans* cells were collected and treated by TRIzol reagent kit (Invitrogen, Carlsbad, CA, USA) and lysed by beads with diameter at 0.1 mm. RNA extraction, reverse transcription, and RT-PCR analysis were then performed described previously (Hu et al. 2021; Kong et al. 2022). Supplemental Table S2 listed all the primer sequences.

### Statistics

One-way ANOVA with Dunnett’s multiple comparison test were employed to analyze the hyphal formation measurement and filipin binding assay. The epithelial cell adhesion and cell damage were analyzed by unpaired *t* test, and two-way ANOVA with Dunnett’s multiple comparison test was employed for the RT-PCR results. All of the statistic analysis was conducted by GraphPad Prism 9 v9.4.1 (GraphPad Software, Beijing, China). The data are reported in the form of mean ± standard deviations (SD) with three biological replicates and three experimental replicates at least.

## Results

### sIgA inhibited the hyphal development and virulence of *C. albicans*

**Fig. 1:**
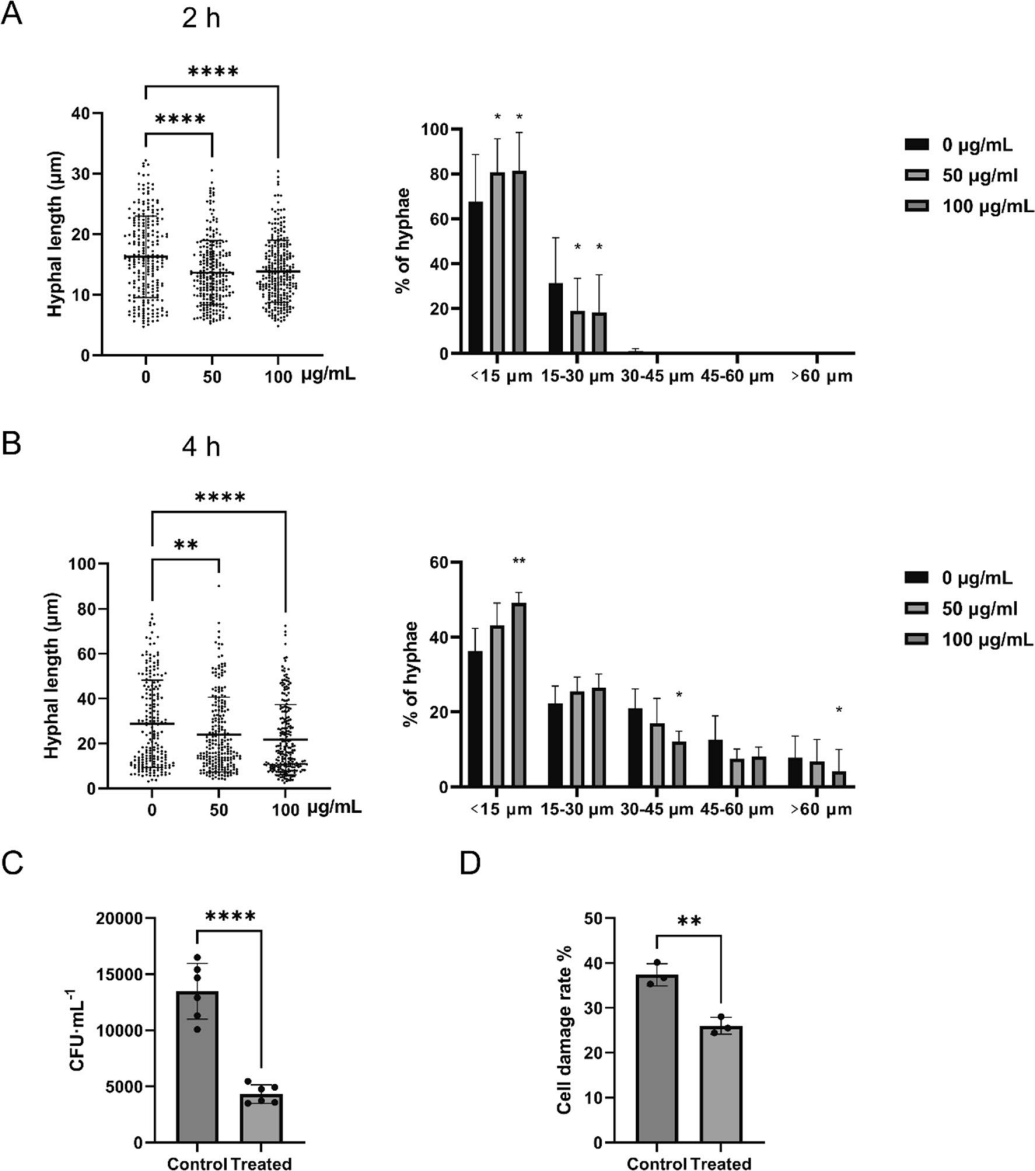
sIgA repressed *C. albicans *hyphal growth and virulence against oral epithelial cells. **A** The hyphal length and distribution of *C. albicans* SC5314 after treatment with by 0, 50, and 100 µg/mL sIgA for 2 h. **B** The hyphal length and distribution of *C. albicans* SC5314 after treatment with 0, 50, and 100 µg/mL sIgA for 4 h. **C** The *C. albicans* SC5314 adhesion to oral epithelial cell HOK with or without 100 µg/mL sIgA. **D** The cell damage of oral epithelial cell HOK caused by *C. albicans* SC5314 with or without 100 µg/mL sIgA. *p < 0.05, **p < 0.01, ****p < 0.0001

First, we measured the direct effects of sIgA against the hyphal growth of *C. albicans* by employing IgG as control. Both 50 and 100 µg/mL sIgA significantly reduced *C. albicans* hyphal length compared to the control groups (0 µg/mL sIgA) at 2 and 4 h (Fig. 1A and B, Supplemental Fig. S1A), while IgG showed no significant effects at the same dosages (Supplemental Fig. S1B, C and D). When calculating the hyphal length distribution, the *C. albicans* showed more yeast or germ tubes (< 15 µm, *p* < 0.05) and pseudohyphal forms (15–30 µm), but less hyphal forms (> 45 µm, *p* < 0.05) treated by both 50 and 100 µg/mL sIgA for 2 h or 4 h (Fig. 1A and B, Supplemental Fig. S1A). IgG showed increase of yeast or germ tubes (< 15 µm, *p* < 0.05) treated by the dose of 100 µg/mL at 2 h (Supplemental Fig. S1C), but both 50 and 100 µg/mL reduced the yeast or germ tubes (< 15 µm, *p* < 0.05) at 4 h (Supplemental Fig. S1D). IgG showed no significant effects on other forms *C. albicans* cells at both 2 and 4 h (Supplemental Fig. S1C and D).

Since *C. albicans* hyphal development is an important virulent factor, its adhesion and damage to oral epithelial cells with or without sIgA were then measured, while IgG was also employed as a control. sIgA significantly reduced *C. albicans* adhesion (Fig. 1C) and cell damage induction (Fig. 1D) to oral epithelial cells indicating that sIgA could block *C. albicans* virulence against host cells by its direct repression on *C. albicans* hyphal development. IgG did not affect *C. albicans* adhesion (Supplemental Fig. S1E), and it even slightly increased the cell damage caused by *C. albicans* (Supplemental Fig. S1F), consistent with the effects of IgG on *C. albicans* hyphal development (Supplemental Fig. S1C and D).

### sIgA affected *C. albicans* ergosterol biosynthesis pathway

**Fig. 2:**
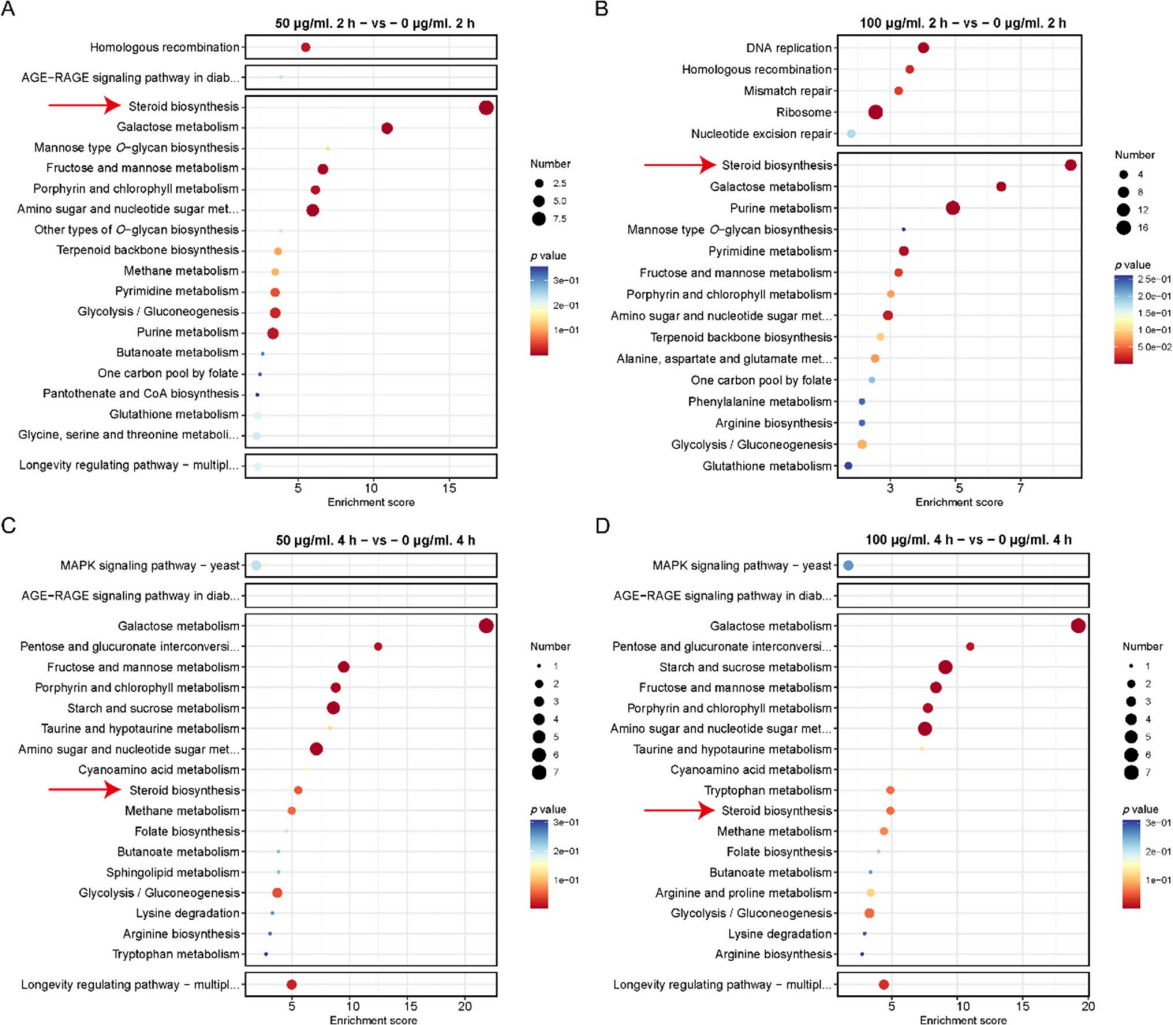
sIgA affected the steroid biosynthesis pathway of *C. albicans*. KEGG enriched pathways (top 20) in *C. albicans* SC5314 treated with 50 µg/ml (**A**) sIgA for 2 h in comparison to the control group. KEGG enriched pathways (top 20) in *C. albicans* SC5314 after treatment with 50 µg/ml (**C**) and 100 µg/ml (**D**) sIgA for 4 h. The steroid biosynthesis pathway was indicated by red arrows

To reveal the mechanisms of sIgA that inhibited *C. albicans* hyphal development and virulence, transcriptomes of *C. albicans* cells treated with sIgA at 50 and 100 µg/mL were sequenced and analyzed. The KEGG pathway enrichment analysis showed that both 50 and 100 µg/mL of sIgA upregulated the steroid biosynthesis pathway at 2 h compared to the untreated group (0 µg/mL of sIgA) (Fig. 2A and B), indicating that sIgA may affect the steroid biosynthesis to inhibit the hyphal development. After the treatment of 50 and 100 µg/mL of sIgA for 4 h, the steroid biosynthesis pathway was also upregulated by sIgA (Fig. 2C and D), further proving the actions of sIgA on the steroid biosynthesis. Then, the differentially expressed genes from the steroid biosynthesis pathway were analyzed; sIgA upregulated most genes of the ergosterol biosynthesis pathway after incubated for 2 and 4 h (Supplemental Fig. S2), indicating that sIgA affected the ergosterol biosynthesis of *C. albicans* to repress its hyphal development.

**Fig. 3:**
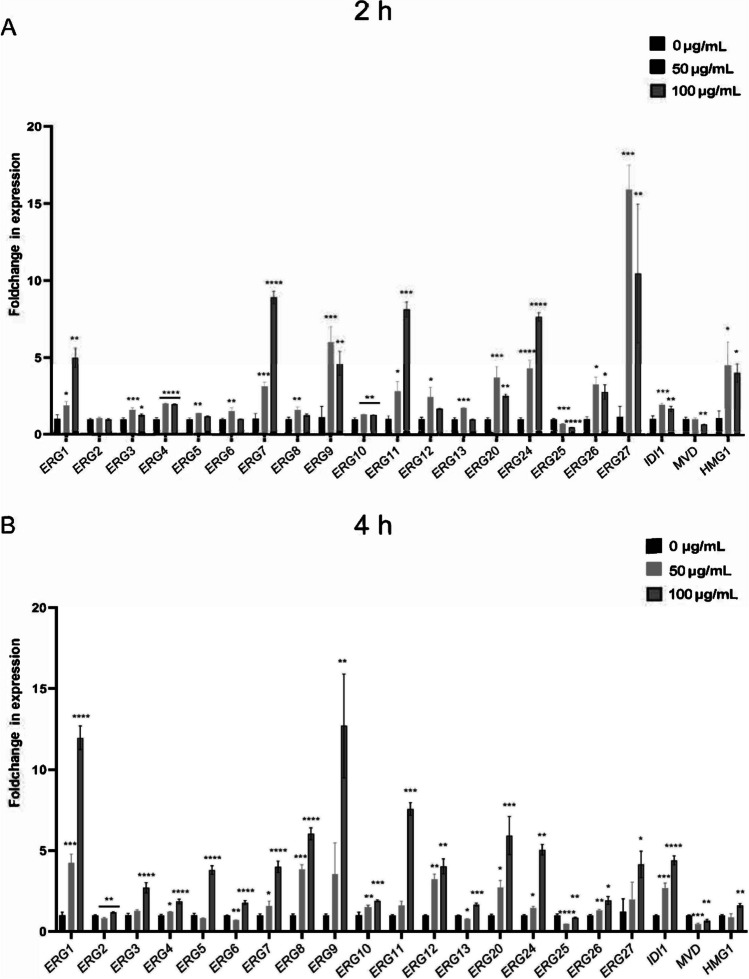
sIgA upregulated the genes expressions from ergosterol biosynthesis pathway. The genes including *ERG1*, *ERG2*, *ERG3*, *ERG4*, *ERG5*, *ERG6*, *ERG7*, *ERG8*, *ERG9*, *ERG10*, *ERG11*, *ERG12*, *ERG13*, *ERG20*, *ERG24*, *ERG25*, *ERG26*, *ERG27*, *MVD*, *HMG1*, and *IDI1* were measured. The experiments were conducted with a minimum of three separate replicates, *p < 0.05, **p < 0.01, ***p < 0.001, ****p < 0.0001, no significance (ns) *p* > 0.05

**Fig. 4:**
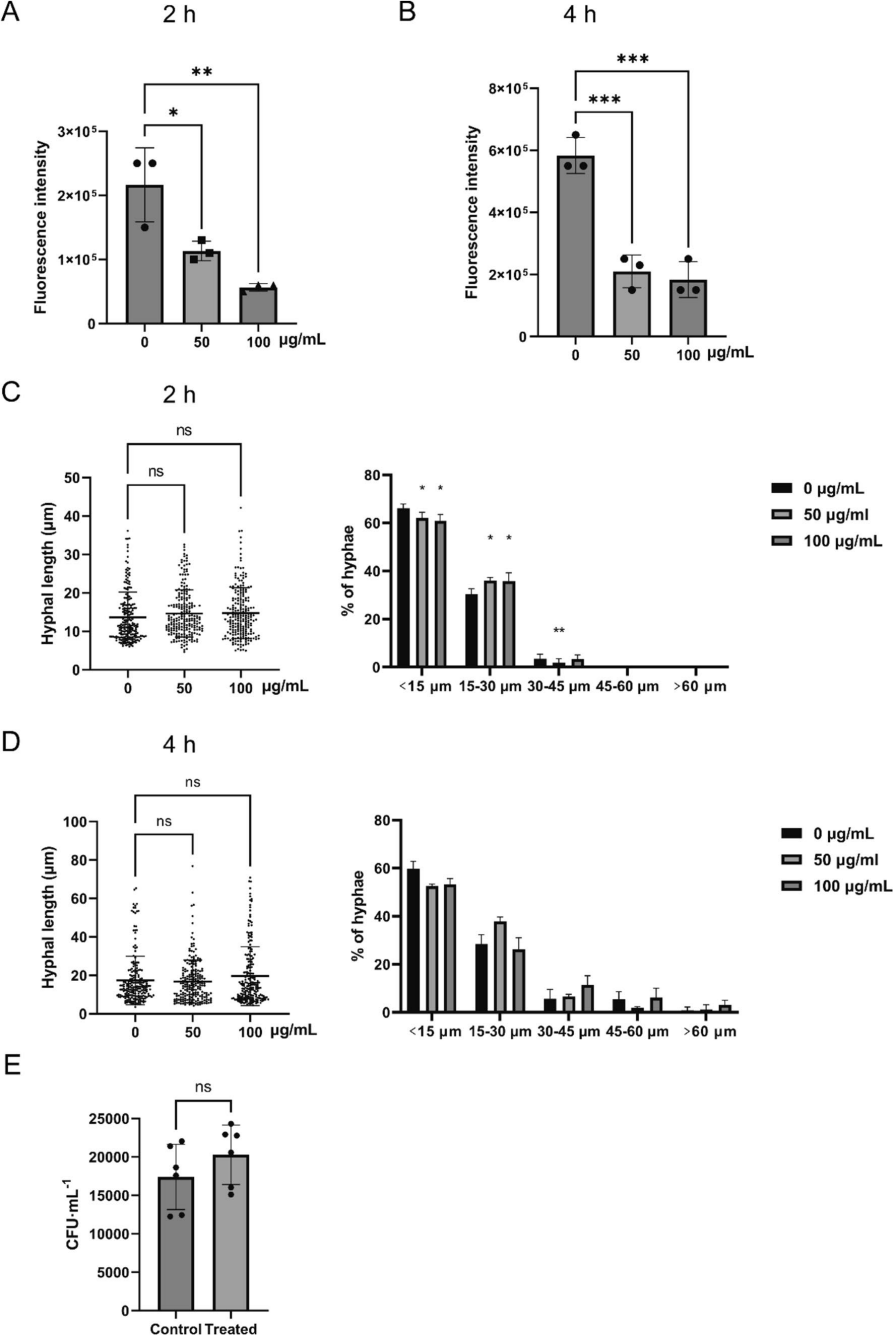
Ergosterol restored the inhibitory abilities of sIgA against* C. albicans* hyphal development and virulence. The ergosterol levels of *C. albicans* cells after treatment with 0, 50, and 100 µg/mL sIgA for 2 h (**A**) and 4 h (**B**). *C. albicans* hyphal length and distribution treated by 0, 50, and 100 µg/mL sIgA with the addition of 10 µg/mL ergosterol for 2 h (**C**) and 4 h (**D**). **E** *C. albicans* adhesion to oral epithelial cell HOK treated by 100 µg/mL sIgA and the addition of 10 µg/mL ergosterol. The experiments were conducted with a minimum of three separate replicates, *p < 0.05, **p < 0.01, ***p < 0.001, no significance (ns) *p* > 0.05

To further confirm the actions of sIgA on the ergosterol biosynthesis, the expressions of 21 genes from this pathway were analyzed by real-time PCR assay (Fig. 3). Most of the genes were upregulated by both 50 and 100 µg/mL of sIgA, including *ERG1*, *ERG3*, *ERG4*, *ERG5*, *ERG7*, *ERG8*, *ERG9*, *ERG10*, *ERG11*, *ERG12*, *ERG20*, *ERG24*, *ERG26*, *ERG27*, *HMG1*, and *IDI1*, while only two genes were downregulated, namely *ERG25* and *MVD* at 2 h (Fig. 3A) and 4 h (Fig. 3B), suggesting that sIgA could directly affect *C. albicans* ergosterol biosynthesis.

### sIgA reduced ergosterol levels of *C. albicans* to inhibit its hyphal development

To identify how sIgA regulated the *C. albicans* ergosterol production, the ergosterol contents treated with sIgA was measured, since the transcriptome analysis suggested that sIgA significantly affected the ergosterol biosynthesis pathway. Both 50 and 100 µg/ml sIgA significantly reduced *C. albicans* ergosterol levels at both 2 (Fig. 4A) and 4 h (Fig. 4B). Then, ergosterol was added to check whether it could affect the hyphal repression activity of sIgA. Both 50 and 100 µg/ml sIgA lost the hyphal development inhibitory activities with the addition of 10 µg/ml ergosterol at 2 (Figs. 4C and S3) and 4 h (Fig. 4D, Supplemental Fig. S3), indicating that sIgA repressed *C. albicans* hyphal development by reducing the ergosterol levels. The addition of ergosterol also reduced the inhibitory abilities of sIgA against *C. albicans* adhesion to oral epithelial cells (Fig. 4E), consistent with that the additional ergosterol restored the hyphal formation inhibited by sIgA.

### sIgA failed to inhibit ERG3 and ERG11 null mutants’ hyphal development and virulence

**Fig. 5:**
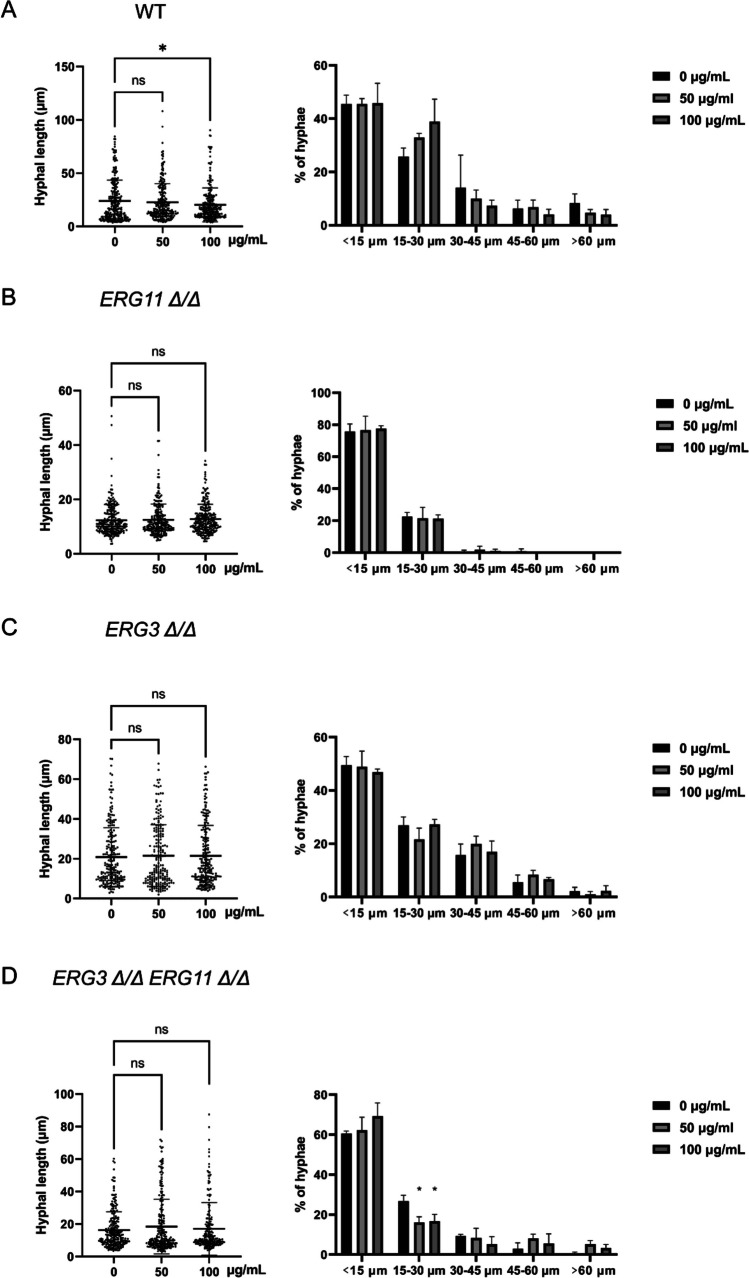
sIgA lost the hyphal development inhibitory abilities on *C. albicans *ergosterol pathway mutants. The hyphal length and distribution of wild-type strain CAF2-1 (**A**), *erg11Δ/Δ* (**B**), *erg3Δ/Δ* (**C**), and *erg3Δ/Δ erg11Δ/Δ* (**D**) treated by 0, 50, and 100 µg/mL sIgA for 4 h. The experiments were conducted with a minimum of three separate replicates, *p < 0.01, no significance (ns) *p* > 0.05

To finally confirm that sIgA repressed *C. albicans* hyphae and virulence through its actions on the reduction of ergosterol levels, *ERG3* and *ERG11* null mutants and their double knockout strain were employed. Both 50 and 100 µg/ml sIgA significantly inhibited the hyphal development of wild type strain (WT) at 2 h (Supplemental Figs. S4A and S5A) and 4 h (Fig. 5A, Supplemental Fig. S4A); however, sIgA lost the hyphal inhibitory activities on the *erg11Δ/Δ* (Fig. 5B and Supplemental Figs. S4B, S5B), *erg3Δ/Δ* (Fig. 5C and Supplemental Figs. S4C, S5C), and *erg3Δ/Δ erg11Δ/Δ* (Fig. 5D and Supplemental Figs. S4D, S5D) mutants at both 2 and 4 h, further indicating that sIgA inhibited *C. albicans* hyphal development through the ergosterol biosynthesis pathway.

**Fig. 6:**
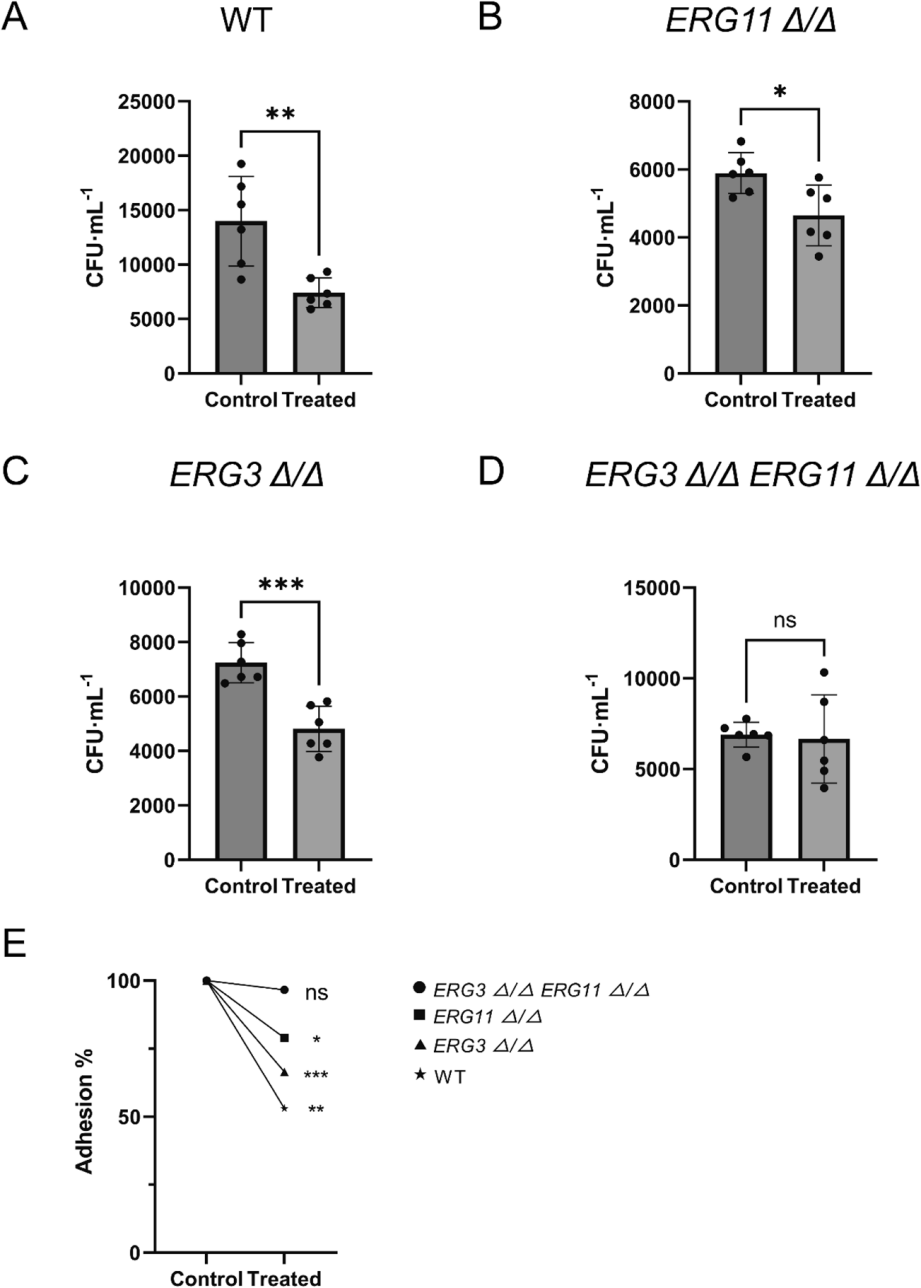
sIgA reduced the virulence inhibitory abilities on *C. albicans *ergosterol pathway mutants. The adhesion to oral epithelial cell HOK of wild-type strain CAF2-1 (**A**), *erg11Δ/Δ* (**B**), *erg3Δ/Δ* (**C**), and *erg3Δ/Δ erg11Δ/Δ* (**D**) treated with 100 µg/mL sIgA. **E** The comparison of the adhesion inhibition rates between strains WT, *erg11Δ/Δ*, *erg3Δ/Δ*, and *erg3Δ/Δ erg11Δ/Δ* with or without the treatments of 100 µg/ml sIgA. The experiments were conducted with a minimum of three separate replicates, *p < 0.05; **p < 0.01; ***p < 0.001; no significance (ns) *p* > 0.05

The adhesions of WT, *erg11Δ/Δ*, *erg3Δ/Δ*, and *erg3Δ/Δ erg11Δ/Δ* strains to host cells with or without sIgA were then measured to check whether ergosterol biosynthesis pathway could affect the inhibitory activities of sIgA on *C. albicans* virulence. sIgA significantly inhibited the adhesion of WT strain to oral epithelial HOK cells (Fig. 6A). sIgA also inhibited the adhesion of *erg11Δ/Δ* (Fig. 6B) and *erg3Δ/Δ* (Fig. 6C) null mutants to oral epithelial cells, but it lost the inhibitory activities on the *erg3Δ/Δ erg11Δ/Δ* double mutant (Fig. 6D). To intuitively represent the different cell adhesion inhibitory abilities of sIgA on these strains, the adhesion inhibition rates compared to the untreated groups were calculated. Compared to the WT strain, sIgA reduced its inhibitory activities of the adhesion of *erg11Δ/Δ* and *erg3Δ/Δ* to oral epithelial cells (Fig. 6E), while sIgA lost the inhibitory activities on *erg3Δ/Δ erg11Δ/Δ* double mutant (Fig. 6E). When compared to *erg11Δ/Δ*, sIgA showed stronger inhibitory abilities on *erg3Δ/Δ* (Fig. 6E), indicating that *ERG11* gene maybe more important than *ERG3* for the actions of sIgA on its inhibition of *C. albicans* hyphal development and virulence.

## Discussion

sIgA is one of the most predominant secretory immunoglobulins (Pabst 2012) and mainly exists at different host mucosa. sIgA has been found to have significant implications to regulate the commensalism of *C. albicans* in the gastrointestinal tract or oral cavity (Doron et al. 2021b; Millet et al. 2020) by its effects on the growth and adhesion to host cells (Moreno-Sabater et al. 2023). However, the mechanisms how sIgA directly acts on *C. albicans* remain unclear. In this study, we found that sIgA directly affected ergosterol biosynthesis pathway of *C. albicans* and reduced the ergosterol contents and then repressed *C. albicans* hyphal development and virulence. sIgA lost or reduced the hyphal and virulence inhibitory capabilities on the key gene mutants from the ergosterol biosynthesis pathway, including *erg11Δ/Δ*, *erg3Δ/Δ*, and *erg3Δ/Δ erg11Δ/Δ* mutants, while the addition of ergosterol restored the activities of sIgA, further indicating the critical roles of ergosterol in the actions of sIgA inhibiting *C. albicans* hyphal development and virulence.

Ergosterol is distributed in various membranes in *C*. *albicans* cells (Schneiter et al. 1999), and it has been proved to play key roles in different cellular progresses, such as maintaining abundance and activity of V-ATPase, ensuring function of ion channels, and assisting biosynthesis of GPI-anchor proteins and the formation of lipid rafts, antifungal drug resistance, and fungal pathogenicity (Lu et al. 2019; Ren et al. 2014; Zhou et al. 2018; Zhu et al. 2021). Our study also demonstrated its key roles in the interactions between sIgA and *C. albicans*, indicating that ergosterol played important roles in *C. albicans* pathogenesis and its response to host immunity. In our previous study, we found the direct effects of ergosterol pathway on the virulence of *C. albicans* as both *ERG11Δ/Δ* and *ERG3Δ/Δ* strains and low dosages of fluconazole significantly reduced the adhesion of *C. albicans* to oral epithelial cells by downregulating the expressions of some adhesion genes (Zhou et al. 2018). sIgA treatment in this study also downregulated the expression of *ALS1*, *ALS2*, and *ALS4* at 2 h, and *ALS1*, *ALS2*, *ALS3*, and *ALS4* at 4 h according to the transcriptome analysis, suggesting that the repression of sIgA on these adhesion genes and virulence could be result from the actions of sIgA on the ergosterol biosynthesis. Meanwhile, *ERG11* was found to play more important roles in the actions of sIgA compared to *ERG3* since sIgA showed stronger inhibitory abilities on *erg3Δ/Δ* than *erg11Δ/Δ*, which might be due to that the deletion of *ERG11* led to the accumulation of toxic lanosterol intermediates and showed more significant effects on the growth and pathogenicity of *C. albicans* (Bhattacharya et al. 2020). However, more details are needed to confirm the importance of all the genes from *C. albicans* ergosterol biosynthesis pathways on the virulence of *C. albicans* and actions of sIgA.

The genes from the ergosterol biosynthesis pathway were significantly upregulated by sIgA in this study. However, the final product ergosterol was significantly reduced, while the additional ergosterol recovered the hyphal development and virulence inhibited by sIgA. sIgA also decreased or failed to represent the inhibitory activities on *ERG3* and *ERG11* null mutants. These results suggested that the upregulation of ergosterol biosynthesis genes might be the result from the feedback regulation of the decrease of cell ergosterol levels. The zinc cluster protein Upc2, coded by *UPC2* gene, is a key regulator to mediate sterol uptake (Shianna et al. 2001) and biosynthesis (Jordá and Puig 2020). In *Saccharomyces cerevisiae*, the hyperactive Upc2 could increase sterol uptake by upregulating the gene *AUS1* and *PDR11* (Wilcox et al. 2002). In *C. albicans*, Upc2 could bind with ergosterol and be located mainly in the cytoplasm under sterol-rich conditions. When the concentration of ergosterol was reduced, Upc2 underwent dissociation from ergosterol and relocated into the nucleus, thereby triggering the expression of ergosterol production genes (Marie et al. 2008; Yang et al. 2015). Upc2 could attach to the promoters of ergosterol biosynthesis genes to induce their expressions (Davies et al. 2005; Germann et al. 2005; Hughes et al. 2000; Vik and Rine 2001; Wilcox et al. 2002). Meanwhile, higher levels of sterol could downregulate *UPC2* to decrease the ergosterol production and lower levels of sterol upregulated *UPC2* to promote ergosterol production (Joshua and Höfken 2017). In our transcriptome and RT-PCR results, the expression of *UPC2* was significantly increased by sIgA, indicating that sIgA reduced the ergosterol levels of *C. albicans* and activated Upc2, then Upc2 upregulated the expressions of the genes from ergosterol biosynthesis pathway. However, the mechanisms of sIgA that reduced the ergosterol levels are still unclear, such as whether sIgA could integrate with ergosterol directly or sIgA could affect the ergosterol transportation, or whether sIgA could block the binding between Upc2 and ergosterol. In addition, overexpression of *UPC2* delayed the elongation of *C. albicans* filaments when subjected to murine macrophages phagocytosis assay (Lohberger et al. 2014), in line with the sIgA repressed the hyphal development of *C. albicans* and upregulated *UPC2* by reducing the levels of ergosterol in our results.

IgA is the major immunoglobulin in saliva, and the concentration of IgA was more than ten times of IgG in healthy individuals’ saliva (Brandtzaeg 2013). Several antigens, including β-glucan and mannan from intestinal *C. albicans*, could also induce IgG response to promote the fungal specific IgG levels in serum (Chiani et al. 2009; Huertas et al. 2017), while *C. albicans* induced IgG also significantly decreased kidney fungal burdens and morality rates in systemic *C*. *albicans* dissemination murine model (Doron et al. 2021a), indicating that IgG has also played key roles against *C. albicans* infections. However, the direct effects of IgG on *C*. *albicans* hyphal development and virulence are unknown. In this study, we found that compared to sIgA, IgG showed no direct effects on the hyphal growth and even slightly increased the virulence to oral epithelial cells, suggesting that *C. albicans* showed different responses to different immunoglobulins to activate various host antifungal immunity.

In conclusion, sIgA repressed *C. albicans* hyphal development and virulence through its direct effects on ergosterol biosynthesis. The findings from our study highlighted the significant role of ergosterol in the pathogenesis of *C. albicans* and the responses of *C. albicans* to host cells and immunological molecules.

## Supplementary Information

Below is the link to the electronic supplementary material.

Supplementary file1 (PDF 1084 KB)

## Data Availability

All data produced or examined in this investigation have been incorporated in this published publication, along with its extra information and accompanying files. The raw data of RNA sequencing was deposited in National Center for Biotechnology Information (NCBI) under the accession number PRJNA1016252.
