# Supplementary material for: Secretory IgA reduced the ergosterol contents of Candida albicans to repress its hyphal growth and virulence
Source: Appl Microbiol Biotechnol. 2024 Feb 29;108(1):244. doi: 10.1007/s00253-024-13063-z (PMC10904422; doi:10.1007/s00253-024-13063-z)
Supplement: Supplementary file 1 — Supplementary file1 (PDF 1084 KB) [file 253_2024_13063_MOESM1_ESM.pdf]

Applied Microbiology and Biotechnology

Secretory IgA reduced the ergosterol contents of *Candida albicans* to repress its hyphal growth and virulence

Jiannan Wang[1#], Jiawei Shen[1#], Ding Chen[1], Binyou Liao[1], Xi Chen[1,2], Yawen Zong[1,2], Yu Wei[1,2], Yangyang Shi[1,2], Yaqi Liu[1,3], Lichen Gou[1], Xuedong Zhou[1,2], Lei Cheng[1,2*], Biao Ren[1*]

1. State Key Laboratory of Oral Diseases & National Center for Stomatology & National Clinical Research Center for Oral Diseases, West China School of Stomatology, Sichuan University, Chengdu 610041, Sichuan, China
2. Department of Operative Dentistry and Endodontics, West China School of Stomatology, Sichuan University, Chengdu 610041, Sichuan, China
3. Department of Pediatric Dentistry, West China Hospital of Stomatology, Sichuan University, Chengdu 610041, Sichuan, China.

# These authors contribute equally to this work.

* Corresponding authors:

Lei Cheng: chenglei@scu.edu.cn

Biao Ren: renbiao@scu.edu.cn

Supplementary figures and tables

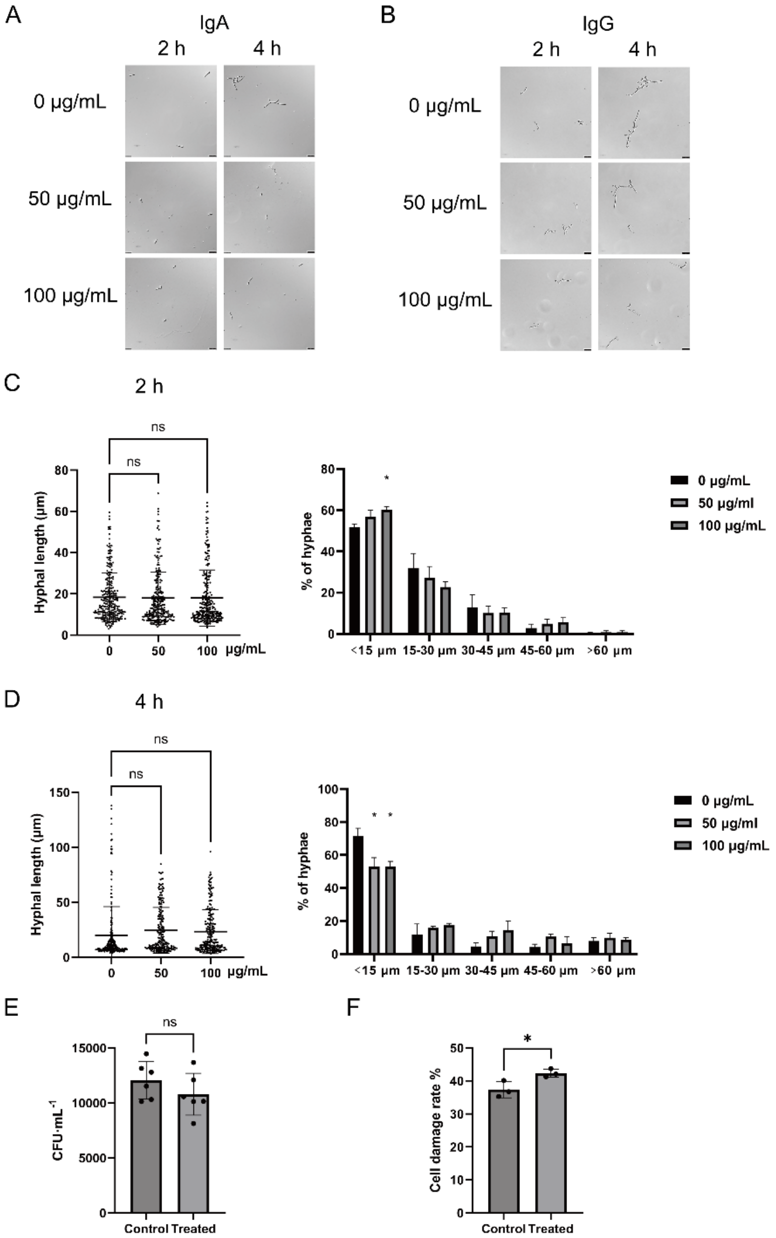

Fig. S1 The hyphal development and virulence inhibition by sIgA and IgG. A. The DIC (differential interference contrast) images of the hyphal formation of *C. albicans* SC5314 treated by 0, 50 and 100 μg/ml sIgA for 2 h and 4 h; **B.** The DIC images of the hyphal formation of *C. albicans* SC5314 treated by 0, 50 and 100 μg/ml IgG for 2 h and 4 h; **C.** The hyphal length and distribution of strain SC5314 treated by 0, 50 and 100 μg/ml IgG for 2 h; **D.** The hyphal length and distribution of strain SC5314 treated by 0, 50 and 100 μg/ml IgG for 4 h; **E.** The *C. albicans*

SC5314 adhesion to oral epithelial cell HOK with or without 100 μg/mL IgG; **F.** The cell damage of oral epithelial cell HOK caused by *C. albicans* SC5314 with or without 100 μg/mL IgG. All of the experiments were performed at least in three distinct replicates and the data are presented as the means ± SD, * $p < 0.05$, no significance (ns) $p > 0.05$.

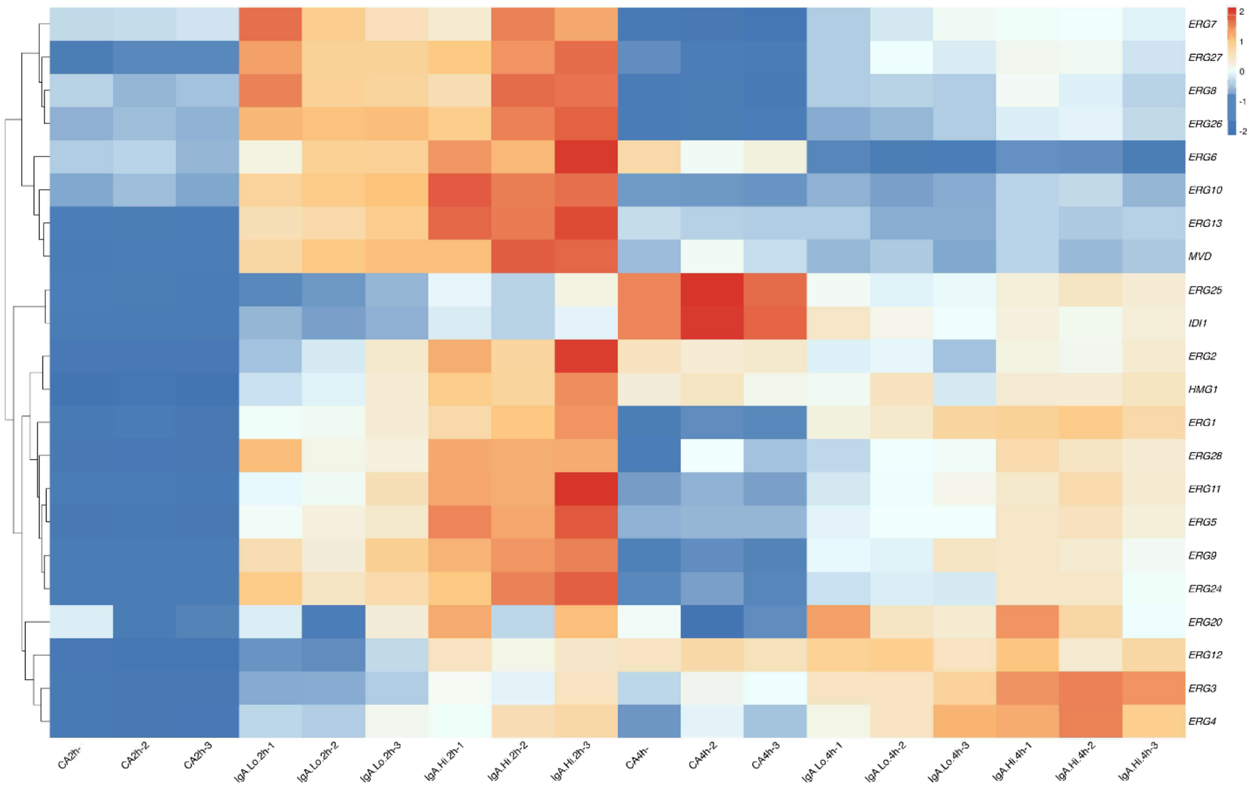

Fig. S2 The expression of genes from the ergosterol biosynthesis pathway. The heatmap indicated the different expressions of the genes from the ergosterol biosynthesis pathway of *C. albicans* treated by 0, 50 and 100 µg/ml sIgA for 2 h and 4 h.

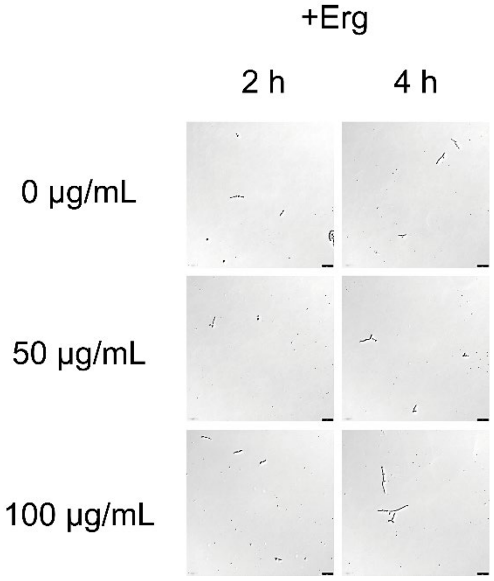

Fig. S3 The addition of ergosterol restored the hyphal development inhibited by sIgA. The DIC images of the hyphal formation of strain SC5314 treated by 0, 50 and 100 μg/ml sIgA for 2 h and 4 h with the addition of 10 μg/ml ergosterol.

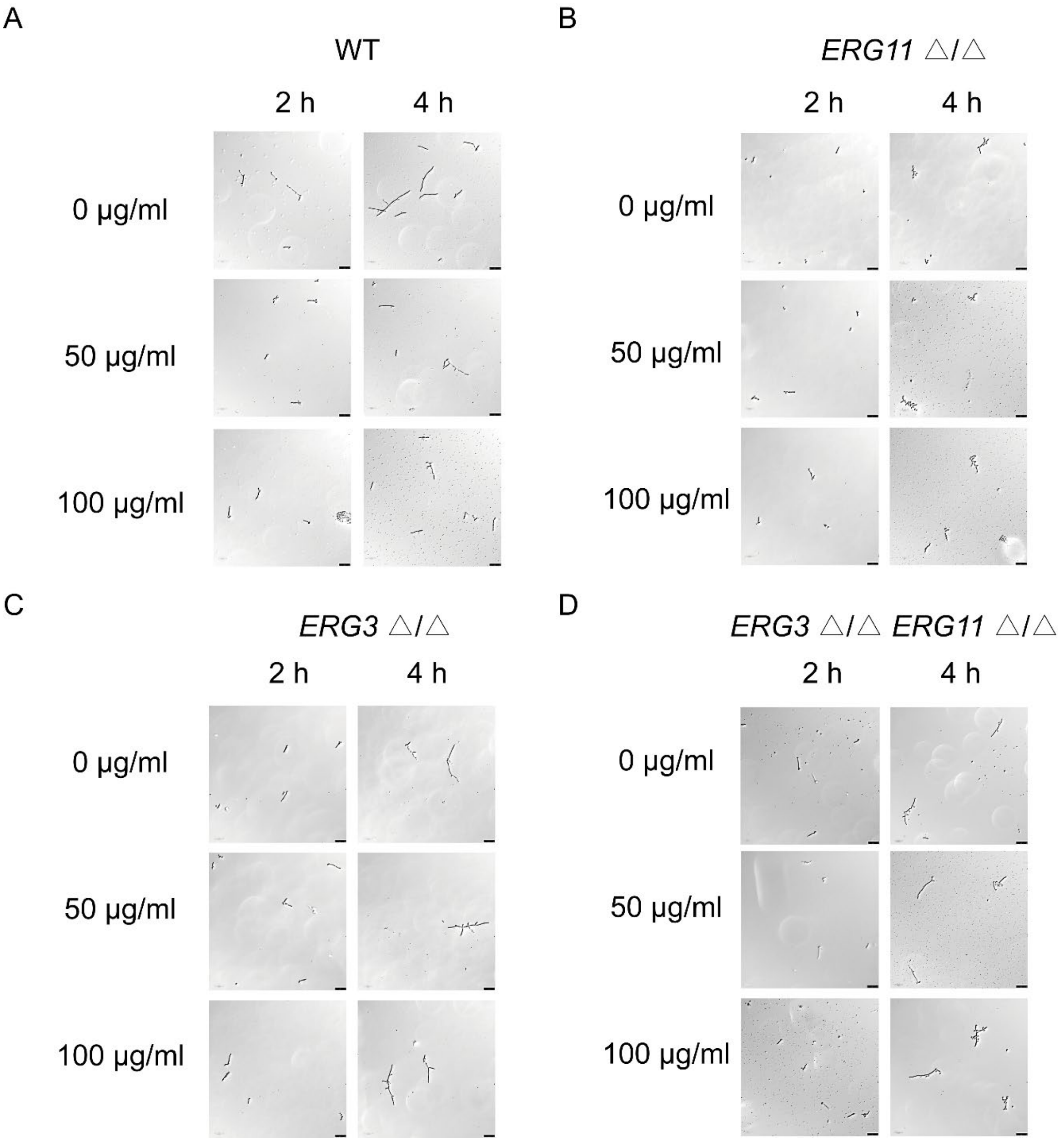

Fig. S4 sIgA lost the hyphal development inhibitory abilities on ergosterol biosynthesis pathway mutants. **A.** The DIC images of the hyphal formation of wild-type strain CAF2-1 treated by 0, 50 and 100 μg/ml sIgA for 2 h and 4 h; **B.** The DIC images of the hyphal formation of *erg11Δ/Δ* treated by 0, 50 and 100 μg/ml sIgA for 2 h and 4 h; **C.** The DIC images of the hyphal formation of *erg3Δ/Δ* treated by 0, 50 and 100 μg/ml sIgA for 2 h and 4 h; **D.** The DIC images of the hyphal formation of *erg3Δ/Δ erg11Δ/Δ* treated by 0, 50 and 100 μg/ml sIgA for 2 h and 4 h.

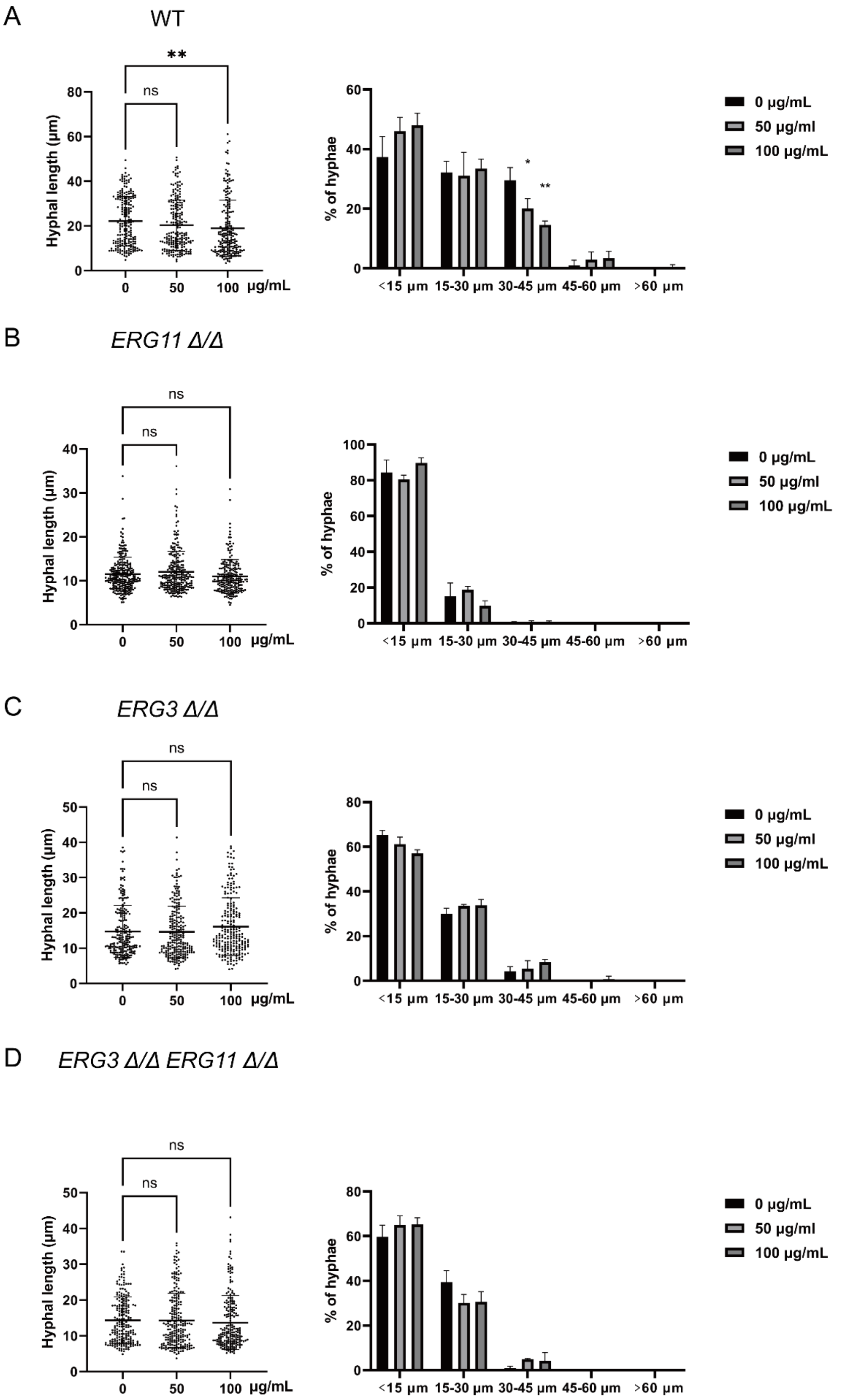
A
WT
**
ns
Hyphal length (µm)
0
50
100 µg/mL
% of hyphae
<15 µm
15-30 µm
30-45 µm
45-60 µm
>60 µm
0 µg/mL
50 µg/ml
100 µg/mL
*
**
B
ERG11 Δ/Δ
ns
ns
Hyphal length (µm)
0
50
100 µg/mL
% of hyphae
<15 µm
15-30 µm
30-45 µm
45-60 µm
>60 µm
0 µg/mL
50 µg/ml
100 µg/mL
C
ERG3 Δ/Δ
ns
ns
Hyphal length (µm)
0
50
100 µg/mL
% of hyphae
<15 µm
15-30 µm
30-45 µm
45-60 µm
>60 µm
0 µg/mL
50 µg/ml
100 µg/mL
D
ERG3 Δ/Δ ERG11 Δ/Δ
ns
ns
Hyphal length (µm)
0
50
100 µg/mL
% of hyphae
<15 µm
15-30 µm
30-45 µm
45-60 µm
>60 µm
0 µg/mL
50 µg/ml
100 µg/mL

Fig. 5 sIgA lost the hyphal development inhibitory abilities on *C. albicans* ergosterol pathway mutants. The hyphal length and distribution of wild-type strain CAF2-1 **(A)**, *erg11Δ/Δ* **(B)**, *erg3Δ/Δ* **(C)**, *erg3Δ/Δ erg11Δ/Δ* **(D)** treated by 0, 50 and 100 μg/mL sIgA for 2 h. The experiments were conducted with a minimum of three separate replicates, * $p < 0.01$, ** $p < 0.01$, no significance (ns) $p > 0.05$

Table S1. *Candida albicans* strains used in this study.

Stain	Genotype	Reference
SC5314	Parent strain, ATCC MYA-2876	(Gillum et al. 1984)
WT[a] CAF2-1	SC5314, *Δura3*::*imm434/URA3*	(Fonzi and Irwin 1993)
erg11Δ/Δ	WT[a], *erg3AΔ*::*hisG/ERG3* *erg11Δ*::*hisG/erg11Δ*::*hisG*	(Sanglard et al. 2003)
erg3Δ/Δ	WT[a], *erg3AΔ*::*hisG/erg3BΔ*::*hisG-URA3-hisG;* *erg11Δ*::*hisG/ERG11*	(Sanglard et al. 2003)
erg3Δ/Δ erg11Δ/Δ	WT[a], *erg3AΔ*::*hisG/erg3BΔ*::*hisG* *erg11Δ*::*hisG/erg11Δ*::*hisG-URA3-hisG*	(Sanglard et al. 2003)

[a]Wild type

Table S2. Real-time PCR primers used in this study.

**Primers**	**Nucleotide Sequence (5'-3')**
18S rRNA	FW-CTAGGGATCGGTTGTTGTTCT
	RV-TTGTGTCTGGACCTGGTGAGT
ERG10	FW-ATCCATGACCAACACGCCAT
	RV-ATTTTTCAGCGGCAACACCC
ERG13	FW-TGCCTTCCATGTGCCAACTT
	RV-GGGACTTGCAAAGCTGGTTG
HMG1	FW-TCGACCATGCGTGGTTGTAA
	RV-TTCATCCCCATGGCATCACC
ERG12	FW-ATTGACAGGTGCTGGAGGTG
	RV-CCTCAATAGCGGCACGATCT
ERG8	FW-TGAGCCTTTGACTGTTGCGA
	RV-ACGGTCCAACAACTGGGTTT

MVD	FW-CCGTCAACATCGCCGTAAGT<br>RV-CACGCTTGAGTACGTGGAGT
IDI1	FW-GGCCAGAAGTTACCCCCTTG<br>RV-GCACGATGTAACAACCCAGC
ERG20	FW-ACCCGTGGCATTAGCAATGTA<br>RV-GACGTTGTTCAGGGGTAGCA
ERG9	FW-ATGGTGTTGCCACCGTTGAA<br>RV-GCAAATTCTTTGGCAGGGGT
ERG1	FW-ATTGGGAGGACATGCACCAG<br>RV-ACTGCATCGTTAGCAGCAGA
ERG7	FW-CTTCCAGACGGTGGATGGTC<br>RV-ACCCTATCAAAGCCCAAGCC
ERG11	FW-ACTCATGGGGTTGCCAATGT<br>RV-AGCAGCATCACGTCTCCAAT
ERG24	FW-ACATGCTGGCTGGCCTATTT<br>RV-GAGAGCTGTCGCTGTTCAGT
ERG25	FW-TGCTGCTCCATTTGGATTGG<br>RV-AATGAGCATCAACGGCTTGG
ERG26	FW-TTTGTTTACGTCCTGCGGGT<br>RV-GTGCTGCCAAAACATGAGCA
ERG27	FW-TTCGTTTGTTGGAGGGGCTT<br>RV-CGCCAGTCCAATCTATGCCT
ERG6	FW-TTGTGGTGTAGGTGGTCCTG<br>RV-TGAACGGTAGCTTCAATGGCA
ERG2	FW-GGTGGTGCCATGGGTACAAT<br>RV-GCACCAGGATAAGCTGCTCT
ERG3	FW-AACGTGCCACTACTGCCATT<br>RV-ACAGATGGCCAGTGTAACCA
ERG4	FW-CCAATGCTTGTGCCAAAGGT<br>RV-ACTGAATGGAACCCCAGCAA
ERG5	FW-TGCTGGTCCCCGTTTCAAAT

RV-AAAGAACGGCGGTAATCGGT

References:

Fonzi WA, Irwin MY (1993) Isogenic strain construction and gene mapping in *Candida albicans*. Genetics 134(3):717-728 doi:10.1093/genetics/134.3.717

Gillum AM, Tsay EY, Kirsch DR (1984) Isolation of the *Candida albicans* gene for orotidine-5'-phosphate decarboxylase by complementation of *S. cerevisiae ura3* and *E. coli pyrF* mutations. Mol Gen Genet 198(2):179-182 doi:10.1007/bf00328721

Sanglard D, Ischer F, Parkinson T, Falconer D, Bille J (2003) *Candida albicans* mutations in the ergosterol biosynthetic pathway and resistance to several antifungal agents. Antimicrob Agents Chemother 47(8):2404-2412 doi:10.1128/aac.47.8.2404-2412.2003
